# Psychological, environmental, and biological factors linked to cognitive decline in Black older adults: a multi‐level approach from the Minority Aging Research Study (MARS)

**DOI:** 10.1002/alz70858_107802

**Published:** 2025-12-26

**Authors:** Juliana Nery Souza‐Talarico, Ana W. Capuano, Lisa L. Barnes

**Affiliations:** ^1^ University of Iowa, Iowa, IA, USA; ^2^ Rush Alzheimer's Disease Center, Rush University Medical Center, Chicago, IL, USA; ^3^ Department of Psychiatry and Behavioral Sciences, Rush University Medical Center, Chicago, IL, USA; ^4^ Rush Alzheimer's Disease Center, Chicago, IL, USA

## Abstract

**Background:**

Black Americans are twice as likely to develop Alzheimer's Disease and Related Dementias (ADRD) compared to White individuals, with psychological and social factors contributing to this disparity. The biological mechanisms linking these factors to cognitive decline are still under investigation. A multi‐domain approach across individual, interpersonal, and community levels is necessary to understand this complex relationship. This study aimed to determine the associations between psychological, environmental, and biological factors linked to cognitive decline in older Black adults.

**Methods:**

Longitudinal data from 118 Black Americans aged 65 and older from the Minority Aging Research Study (MARS) were analyzed. The biological factors at the individual level included blood‐based neuroendocrine (cortisol, DHEA‐S, testosterone, IGF‐1), immunologic (IL‐10, IL‐1ra, IL‐6, CRP, TNF‐α), and metabolic (LDL‐C, HbA1C, BMI, GFR) markers. Discrimination, a psychological factor at the interpersonal level, was measured using the Everyday Discrimination Scale (EDS). The Social Vulnerability Index, a geospatial data comprising socioeconomic, housing and transportation, minority groups and language, and household and disability, assessed the environmental factors at the community level. Cognitive decline, the primary outcome, was evaluated using annual performance on global cognition, processing speed, working, semantic, and episodic memory tests since 2004. Ordinal models and a stepwise approach identified markers associated with discrimination, social vulnerability, and cognition.

**Results:**

Controlling for sex, age, and education, high EDS was associated with neuroendocrine (cortisol *p* = .007; IGF‐1 *p* = .026; testosterone *p* = .009) and immunological markers (IL‐1ra *p* = .006; TNF‐α *p* = .044), while high SVI was linked to neuroendocrine (cortisol *p* = .0010; IGF1 *p* = .018), and metabolic markers (LDL *p* = .005, BMI *p* = .044). In the final models, EDS (*p* = .039), SVI (*p* = .019), neuroendocrine (IGF1 *p* = .029, DHEAS *p* = .022), immunological (CRP *p* = .016; IL‐1ra *p* = .05; TNF‐α (*p* = .030) and metabolic (HbA1C *p* = .018 BMI *p* = .03, glucose *p* = .038, GRF1 *p* = .029) markers were associated with decline in global cognition, working memory processing speed and semantic memory.

**Conclusion:**

These findings suggest that psychological, environmental, and biological factors at the individual, interpersonal, and community levels may synergistically contribute to cognitive decline. Our findings inform future research incorporating ADRD biomarkers to improve early prediction of ADRD and enable timely prevention through precise medicine.

